# TB Treatment Delays in Odisha, India: Is It Expected Even after These Many Years of RNTCP Implementation?

**DOI:** 10.1371/journal.pone.0125465

**Published:** 2015-04-30

**Authors:** Kumaravel Ilangovan, Sharath Burugina Nagaraja, Ramya Ananthakrishnan, Anil G. Jacob, Jaya Prasad Tripathy, Deepak Tamang

**Affiliations:** 1 Health Systems Research India Initiative, Thirvananthapuram, India; 2 Department of Community Medicine, ESIC Medical College and PGIMSR, Bangalore, India; 3 Resource Group for Education and Advocacy for Community Health (REACH), Chennai, India; 4 International Union Against Tuberculosis and Lung Disease (The Union), South-East Asia Office, New Delhi, India; 5 Department of Community Medicine, School of Public Health, PGIMER, Chandigarh, India; The Foundation for Medical Research, INDIA

## Abstract

**Background:**

In India, the Revised National TB Control Programme (RNTCP) envisages initiation of TB treatment within seven days of diagnosis among smear-positive patients. After nearly two decades of RNTCP implementation, treatment delays are usually not expected.

**Objectives:**

To determine the proportion of sputum smear-positive TB patients who were initiated on treatment after seven days and their associated risk factors.

**Methods:**

The study was conducted in Cuttack and Rayagada districts of Odisha. It was a retrospective cohort study that involves review of TB treatment registers and laboratory registers for 2013.

**Results:**

Among 1,800 pulmonary TB (PTB) patients, 1,074 (60%) had been initiated on treatment within seven days of diagnosis, 721 (40%) had been initiated on treatment more than seven days, and 354 (20%) had delays of more than 15 days. The mean duration between TB diagnosis and treatment initiation was 21 days with a range of 8–207 days (median = 14 days). Odds of treatment delay of more than seven days were 4.9 times (95% confidence interval [CI] 3.3-6.6) among those who had been previously treated, 6.2 times (95% CI 1.3-29.7) among those infected with HIV, and 1.8 times (95% CI 1.1-2.9) among those diagnosed outside district DMC.

**Conclusion:**

Delay in initiation of TB treatment occurred in majority of the smear-positive patients. The RNTCP should focus on core areas of providing quality TB services with time-tested strategies. To have real-time monitoring mechanisms for diagnosed smear-positive TB patients is expected to be the way forward.

## Background

Tuberculosis (TB) still remains a major global public health problem; according to the WHO Global TB report and TB India report (2014) globally, there are 8.6 million incidence cases out of which 2.3 million (26%) are in India. The infectious sputum smear positive cases are estimated to be 2.5 million incidence cases globally out of which 0.9 million (37%) are diagnosed in India[1,2]. It is imperative that all the diagnosed TB cases to be initiated on TB treatment at the earliest possible time.

In India, the Revised National TB Control Programme (RNTCP) is being implemented since 1997, with complete country coverage from 2006 onwards. The programme has consistently achieved the global objectives of 70% case detection and 85% cure rate from 2007 onwards [3]. The RNTCP envisages initiation of TB treatment within seven days of TB case detection [4]. In practice, delays have been observed in treatment initiation and this varies across states in India [5,6].

In India’s federal system, health is a subject for which individual states in India are responsible; the implementation of any national health programme depends on the efficiency of the general health system in various states. One of the important indicator for the quality of Directly Observed Treatment Short course (DOTS) implementation under RNTCP is the proportion of smear-positive TB patients initiated on treatment within seven days of diagnosis [4]. In 2014, nationally 88% of patients registered with smear-positive PTB were initiated on DOTS treatment within seven days of diagnosis. This figure ranges from 30–100% across the districts in the country and in some districts, as few as 5% of patients were initiated on treatment within seven days [2]. Studies conducted at Nalgonda district, Andra Pradesh and Bardhaman district, West Bengal, India in 2010 had found that 35% of patients had greater than seven days of treatment delay under RNTCP settings [7]; it is presumed that after three years of this study and nearly 20 years of RNTCP implementation, strategies and mechanisms to avoid any treatment delays are well in place.

We planned this study in one of the least developed state of India, Odisha, [8] to determine the treatment delay for smear positive TB patients registered under RNTCP in Cuttack and Rayagada districts. Both the selected districts had recorded poor performance for timely initiation of treatment consistently from 2009 onwards. The proportion of smear positive who had timely initiation during the period between 2009and2012 for (a) Cuttack district was—79%, 79%, 78%, 77%, respectively for these years; (b) similarly, for Rayagada district—the proportion of smear-positive patients who had timely initiation of treatment for these years was 78%, 74%, 69%, 74%, respectively for these years [2,9–11]. There were no studies from the region of Odisha on treatment initiation delays. Each state and district in our country is unique. The factors in other regions may not be necessarily applicable to the all other regions. This study intends to bring in a better understanding about the delays in TB treatment initiation and their associated risk factors.

### Objectives

To determine amongst all RNTCP smear positive TB patients registered at Cuttack and Rayagada districts, Odisha in the year of 2013,

1. The number and proportion of TB patients who experienced a treatment delay of more than seven days
2. The associated risk factors for patients who experienced a treatment delay of more than seven days.

## Materials and Methods

### Study design

We used a retrospective cohort study design that involved review of RNTCP TB treatment registers and laboratory registers of Cuttack and Rayagada district, Odisha, 2013.

### Study setting

The state of Odisha has a population of 41 million and is located at the eastern coast of India adjacent to Bay of Bengal. Administratively, it is divided into three divisions namely north, central, and south; that includes 30 districts, 58 sub-divisions, 317 *tahsils* and 314 blocks [12,13].

Geographically, the Rayagada and Cuttack district located at mountainous hilly terrain and coastal plain areas respectively. RNTCP has been implemented in Rayagada and Cuttack district since 2001 and 2004 respectively [14]. The Rayagada district has a population of 0.96 million with three tuberculosis units (TU) and 21 designated microscopy centers (DMC), 188 peripheral health institutions (PHI). Cuttack district has a population of 2.6 million with 5 TUs, 26 DMCs and 371 (PHI). All the PHI and DMCs at both districts had functional DOT centers. In 2013, 1674 and 1518 TB patients were initiated on treatment at Rayagada and Cuttack districts respectively.

In India after diagnosis, all patients report to their local area DOT Centers to start treatment where drugs are provided to the TB patients in patient wise boxes to ensure that all drugs for full course of treatment are earmarked on the day one, a patient is registered for treatment under the programme. DOT centers are highly decentralized currently more than 400,000 DOT centers have been established near to residence of patients to the extent possible. They serve as treatment delivery point from where a DOT provider operates (e.g. home of a village health worker, community volunteer, local sub-centre where a nurse or Accredited Social Health Activists (ASHA) operates, Anganwadi Worker (AWW)) in addition NGOs, private practitioners also involved under the RNTCP to serve as DOT center/provider. The DOT provider covering the area of the DOT Centre collects the anti-TB medication from the PHI, conducts an Initial Home visit (IHV) for verifying the address, and counsels the patient. She/he then identifies a DOT Provider for the patient and starts the patient on treatment [7,15].

### Study population

All sputum smear positive TB patients registered at Cuttack and Rayagada districts of Odisha for the year2013.

### Variables and data collection, data analysis and statistics

#### Source of data and collection

Data were collected from the TB treatment registers (January-December 2013) available in the TU’s and Laboratory registers available in the Designated Microscopy Centre (DMC’s) of the respective districts from February—April 2014 in a structured paper based data collection forms by the principle investigator (PI). Personally identifiable information like name, address was not recorded hence the data was anonymous. Variables collected from TB treatment registers included age, sex, date of initiation of treatment, date of registration, date of diagnosis, DMC name, smear status, type of TB, HIV status and category of anti- TB treatment. Laboratory registers were used to verify the smear status and date of diagnosis by tracking through laboratory number registered in TB register. If the date of diagnosis was different in laboratory register from that of TB treatment register, date from the laboratory registers has been recorded for the study. We used EpiData software (version 3.1) for data entry and Statistical Package for Social Sciences (SPSS) version 16.0 for analysis. Data were double entered into an EpiData database by two independent data entry operators and validated. The two data bases were compared for consistency and all inconsistencies were resolved by referring to the original data collection sheet.

#### Data analysis

The number of days between the date of diagnosis of PTB and the date of initiation of treatment was collected from the TB registers and the laboratory register. The outcome variable was delayed treatment initiation, defined as treatment starting after seven days of TB diagnosis. Exposure variables included: age, sex, health system level of microscopy facility [TB Unit (TU-DMC), DMC of other districts, Medical College DMC, DMC of other TU’s and other Peripheral Health Institution (PHI-DMC)], type of TB [new smear positive-NSP, retreatment—RT], HIV status and presence of microscopy at the health facility where the patient was diagnosed (PHI with or without microscopy).

Crude and adjusted odds ratios were calculated with 95% confidence limits to assess the relation between these categorical variables and delayed initiation of treatment. We determined a P-value of <0.05 was statistically significant and all tests of significance were two-tailed. Independent variables which were significantly associated with our outcome of interest during the crude analyses were all included in one logistic regression model to calculate adjusted associations with 95% confidence intervals.

### Ethical considerations

Since this study involved the review of records routinely collected by the national programme and did not involve any patient interactions, informed consent was not needed. Data was entered in a designed format based on the information recorded in the registers. Personally identifiable information like name and address was not recorded hence the data was anonymous. Data was securely maintained by keeping data collection forms in a lockable cabinet; the electronic data file kept in a password protected computer accessible only to the principal investigator.

Ethical approval was obtained from the Institutional Review Board of Asian Institute of Public Health, Bhubaneswar, Odisha and the Ethics Advisory Group of the International Union against Tuberculosis and Lung Disease, Paris, France. Due permissions were obtained from the State Tuberculosis Officer (STO) and the Director of Health Services Odisha to access records in the study districts.

## Results

A total of 3192 TB patients had registered for TB treatment, out of which 1805 (56%) were smear positive pulmonary and 1387 (44%) smear negative and extra pulmonary TB cases Among the smear positive patients, majority (75%) were males with a median age of 40 years. Nearly 40% (721) of these patients had encountered a treatment delay of more than seven days and this included 354 (20%) with treatment delay of more than 15 days [Table 1]. The mean number of days for treatment initiation was 21 (8–207) days and median was 14 days.

**Table 1:** Time taken to initiate TB treatment among smear positive TB patients, Cuttack and Rayagada district Odisha, 2013.

Number of days	Number of patients (%)
**0 to 7**	1079 (59.8)
**8 to 14**	367 (20.3)
**15 to 25**	174 (9.6)
**Above 25**	180 (10)
**Total**	1800 (100)

[Table 2] Odds of delayed initiation of treatment were found to be more in the retreatment cases 4.9 times (95% CI 3.3–6.6) than the new cases. There was a significant delay amongst 6.2 times (95% CI 1.3–29.7) those infected with HIV and 1.8 times (95% CI 1.1–2.9) among those diagnosed outside district DMC. The HIV status was known in 27% of PTB cases only.

**Table 2:** Associated risk factors for delay in treatment initiation among smear positive TB patients, Cuttack and Rayagada district, 2013.

Risk factors	Initiation of treatment <7 days	Initiation of treatment >7 days	Unadjusted odds ratio	P-value	Adjusted odds ratio	P-value
Age groups	n (%)	n (%)	(95% CI)	(<0.05)	(95% CI)	(<0.05)
(in years)	n = 1079	n = 721				
**0–14**	23 (49)	24 (51)	2.0 (1.0–4.0)	0.06	1.8 (0.8–3.7)	0.1
**15–34**	408 (63)	237 (37)	1.1 (0.7–1.7)	0.6	1.1 (0.7–1.7)	0.7
**35–54**	438 (59)	300 (41)	1.3 (0.8–2.0)	0.2	1.2 (0.8–1.9)	0.3
**55–64**	142 (53)	124 (47)	1.6 (1.1–2.6)	0.3	1.6 (1.1–2.6)	0.05
**65 & above**	68 (65)	36 (35)	Reference			
Sex						
**Male**	807 (59)	552 (41)	1.1 (0.9–1.4)	0.4		
**Female**	272 (62)	169 (38)	Reference			
Type of case						
**Retreatment cases**	77 (28)	199 (72)	4.9 (3.7–6.5)	<0.001	4.9 (3.7–6.6)	<0.001
**New cases**	1002 (66)	522 (34)	Reference			
HIV status						
**Positive**	3 (27)	8 (73)	6.8 (1.5–30.4)	0.01	6.2 (1.3–29.7)	0.02
**Negative**	307 (64)	177 (36)	1.5 (0.7–3.1)	0.3	1.4 (0.6–2.9)	0.4
**Unknown**	28 (72)	11 (28)	Reference			
**Not recorded**	742 (59)	525 (41)				
Type of diagnostic facility						
**Medical college**	150 (59)	105 (41)	1.0 (0.8–1.4)	0.7	1.1 (0.8–1.5)	0.7
**Outside district DMC**	34 (42)	47 (58)	2.1 (1.3–3.3)	<0.001	1.8 (1.1–2.9)	0.02
*TU-DMC	275 (64)	152 (36)	0.8 (0.6–1.0)	0.1	0.9 (0.7–1.1)	0.2
**Outside TU-DMC**	63 (58)	45 (42)	1.1 (0.7–1.6)	0.7	1.1 (0.7–1.7)	0.6
**Other PHI-DMC**	557 (60)	372 (40)	Reference			
Microscopy in treating unit						
**Without microscopy**	588 (59)	410 (41)	0.9 (0.8–1.1)	0.4		
**With microscopy**	491 (61)	311 (39)	Reference			

*TU-Tuberculosis unit, DMC-Designated microscopy centre, PHI-Peripheral health institutions

## Discussion

This is the first study conducted in the state of Odisha, India to determine the delays in TB treatment initiation and their associated factors under programmatic settings. The findings revealed that two in five patients diagnosed with smear positive TB experienced a delay of more than seven days while one in five patients experienced delay of more than 15 days for treatment initiation. The main factors associated with delay were found to be patients with history of prior treatment and those diagnosed at outside the district DMCs and patients with HIV co-infection. Only 60% of smear positive PTB patients were initiated on treatment within seven days of diagnosis whereas the state average was 84% and national average was 88% for year 2013 [2].

The study findings have the following implications, First, the duration between TB diagnosis and treatment initiation in our study ranged from 8–207 days when compared to a study conducted at Nalgonda and Bardhaman districts where in it was found to be 0–128 days under programmatic settings [7]. The increase in delay in our study could be probably attributed to the weak general health system and the difficult terrain with lack of adequate transport facilities for the patients and provider to access and deliver the timely health services [16].

This range of time is too wide with clear epidemiological implications, and merits closer policy-level examination. The annual new smear positive TB (NSP) case notification rate per lakh population for Rayagada district was 86 (2011), 92 (2012), 95 (2013) which shows increasing trend and for Cuttack district annual NSP case notification was 24 (2011), 24 (2012) and 20 (2013) which shows stagnation in case finding [2,3,11]. This trend could be attributed to delayed treatment initiation among infectious smear positive cases and poor utilization of TB care services respectively.

Second, the associated risk factors for delays in our study were patients with a history of previous TB treatment and those diagnosed outside the district DMCs. A patient with a history of prior TB treatment requires a category II regimen which includes an injection to be provided on alternate days for a period of two months. Much time would have been probably lost by the TB health staff in identifying a DOT provider and a nearby practitioner to provide injections regularly. Further, patients who have had prior DOT may have experienced side effects of drugs, so inadequate timely supportive treatment for the side effects could be the reason for reluctant to start category II treatment. This could be addressed through ensuring adequate attention to side effects, timely referral by DOT providers and proper management by Medical Officers [16].

Third, the delay among patients who were diagnosed outside the district DMCs was due to the sub-optimal referral system which requires the need of a referral to a district microscopy centre for testing and loss of time for referral back to the local health system for initiation of treatment. Patients who are diagnosed outside their own area have an increased likelihood of delay. Currently, the RNTCP has adhered to a mechanism of paper-based referral and feedback system when a patient is diagnosed outside his or her area. This process is time consuming. This could be addressed through decentralization of NIKSHAY case based-web based notification system to the DMC’S and PHIs so that every patient diagnosed as TB will be notified immediately and referred back to the referral units. Despite service availability, some patients seek care outside their local area; this was not addressed in this study. A previous study done in Chennai and the Madurai districts of Tamil Nadu, India on health seeking behaviour of persons with symptoms typical of pulmonary TB had suggested that patient do not seek health care services at certain centres that are not patient friendly [17].

This could be addressed by making health care facilities are more approachable and accessible to help patients more use of these facilities. Transportation of anti TB-drugs for a diagnosed patient to a local DOT centre after diagnosis may contribute to delay; this could be addressed through decentralization of treatment stocks and providing incentives to community DOT providers to provide timely DOT initiation.

Fourth, as per the RNTCP’s policy all the TB patients should be screened for HIV. However, it was observed that only 27% of the PTB cases knew their HIV status which is a serious concern. It may be due to the poor implementation of HIV-TB collaborative activities in the area; immediate measures are to be taken to ensure that HIV counselling and testing centres are co-located at the same health facility [18].

## Conclusion and Recommendations

To conclude, even after two decades of RNTCP implementation, significant proportion of smear positive TB patients have treatment initiation after seven days. Complacency in implementation of basic TB services risks seriously affecting on-going TB control efforts in the country. The RNTCP should strive to have real time monitoring mechanism for diagnosed smear positive TB patients and reconsider measures to avoid treatment delays; with special focus on effective implementation of social action plan where required (including the tribal action plan) [19].

## Strengths and Limitations

The strength of this study was that it included all the TB patients registered in the districts under programmatic settings which actually reflect ground realities. The continuous supervision and monitoring of the records by the programme staff would have made the data more reliable. STROBE guidelines have been followed in this study [20]. The data was double entered and validated to ensure data quality and to avoid transcription errors. The study has the following limitations: (a) We could only assess variables which are routinely collected in the registers, records and reports; there might be other factors related to delay which might have been missed. (b) We could not differentiate between patient-related delays and provider-related delays from the register reviews [7]. (c) As this is a retrospective analysis, factors associated with treatment delays are conjectural and should be viewed in that perspective.
